# National rare diseases registry in Spain: pilot study of the Spanish Rare Diseases Registries Research Network (SpainRDR)

**DOI:** 10.1186/1750-1172-9-S1-P5

**Published:** 2014-11-11

**Authors:** Verónica Alonso, Ignacio Abaitua, Óscar Zurriaga, Jenaro Astray, Manuel Errezola, Josefa M Aldana-Espinal, Mario J Margolles, Josep Jiménez, Joaquín A Palomar, Milagrosa Santana, Enrique Ramalle-Gomarra, Julián M Ramos, Federico E Arribas, Rufino Álamo, Gonzalo Gutiérrez-Ávila, Antònia Galmés, Miguel García-Ribes, Carmen Navarro, Eva Ardanaz, Manuel Posada de la Paz

**Affiliations:** 1 Institute of Rare Diseases Research, Instituto de Salud Carlos III, CIBERER, Madrid 28029, Spain; 2 DG Salud Pública, FISABIO-Saud Pública, CIBERESP, Valencia 46020, Spain; 3 Subdirección de Promoción de salud y Prevención, Consejería de Sanidad, 28037 Madrid, Spain; 4 Departamento de salud, Gobierno Vasco, Vitoria-Gasteiz 01010, Álava, Spain; 5 Servicio andaluz de Salud, Sevilla 41071, Spain; 6 DG Salud pública, Consejería de Sanidad, Oviedo 33006, Asturias, Spain; 7 Servei Català de la Salut, Barcelona 08018, Spain; 8 Consejería Sanidad y Política Social, Servicio Planificación y Financiación Sanitaria, Murcia 30001, Spain; 9 Scio. Pediatría, C. Hospitalario Univ. Insular Materno Infantil, Las Palmas de Gran Canaria 35016, Spain; 10 Servicio Epidemiología y Prevención Sanitaria, Gobierno de La Rioja, Logroño 26071, La Rioja, Spain; 11 Subdirección de Epidemiología, Servicio Extremeño de Salud, Mérida 06800, Badajoz, Spain; 12 DG Planificación y Aseguramiento, Servicio Evaluación y Acreditación Sanitaria, Zaragoza 50017, Spain; 13 Observatorio de Salud Pública, DG Salud Pública, Valladolid 47071, Spain; 14 DG Salud Pública, Drogodep. y Consumo, Consejería Sanidad y Asuntos Sociales, Toledo 45005, Spain; 15 DG Salut Pública i Consum, Conselleria Salut, Palma 07010, Islas Baleares, Spain; 16 Scio Atención Sanitaria, Consejería Sanidad y Servicios Sociales, Santander 39009, Cantabria, Spain; 17 Scio. Anatomía Patológica, Instituto de Investigación Biomédica de Vigo, Vigo 36200, Pontevedra, Spain; 18 Instituto de Salud Pública de Navarra, CIBERESP, Pamplona 31003, Navarra, Spain

## Background

The development of a national Rare Diseases (RD) registry in Spain was launched in 2012 with the project SpainRDR, supported by the International Rare Diseases Research Consortium (IRDiRC). SpainRDR includes two different strategies: patient registries addressed to patient outcome research and population-based registries addressed to epidemiologic research, health and social planning [1]. The pilot study aims to detect the difficulties of developing the national and population-based RD registry.

## Material and methods

Both comprehensive RD lists and common data elements (CDE) have been defined and harmonized with other international strategies (EPIRARE, RD-CONNECT, NIH). CDEs mainly comprise variables related to personal identification data and RD definition. RD patient information was collected from regional health databases corresponding to 2010 and 2011: electronic hospital records (discharges basic minimum dataset), mortality registry, health insurance card databases, electronic primary care clinical records, chronic renal diseases registry, orphan drugs registry, newborn screening registry and tumor registry, among others.

## Results

**Figure 1:**
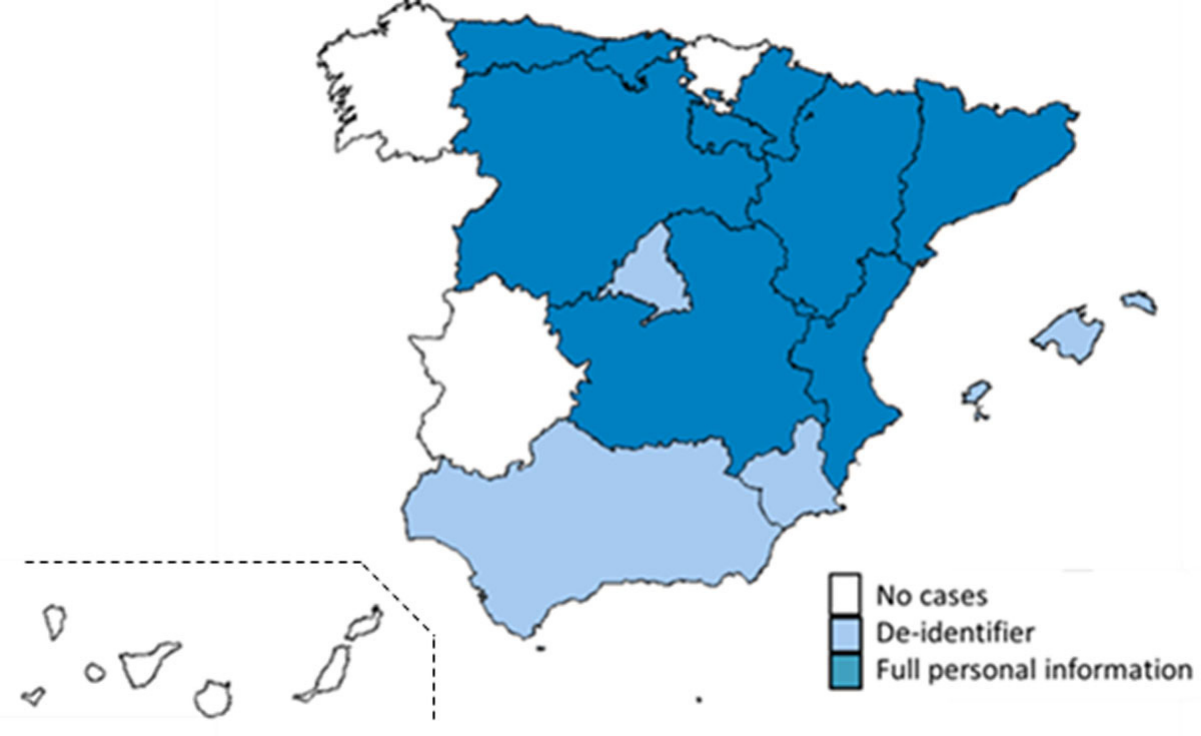
Participation of Regional Health Departments in the pilot study: types of reported cases to central repository

**Figure 2:**
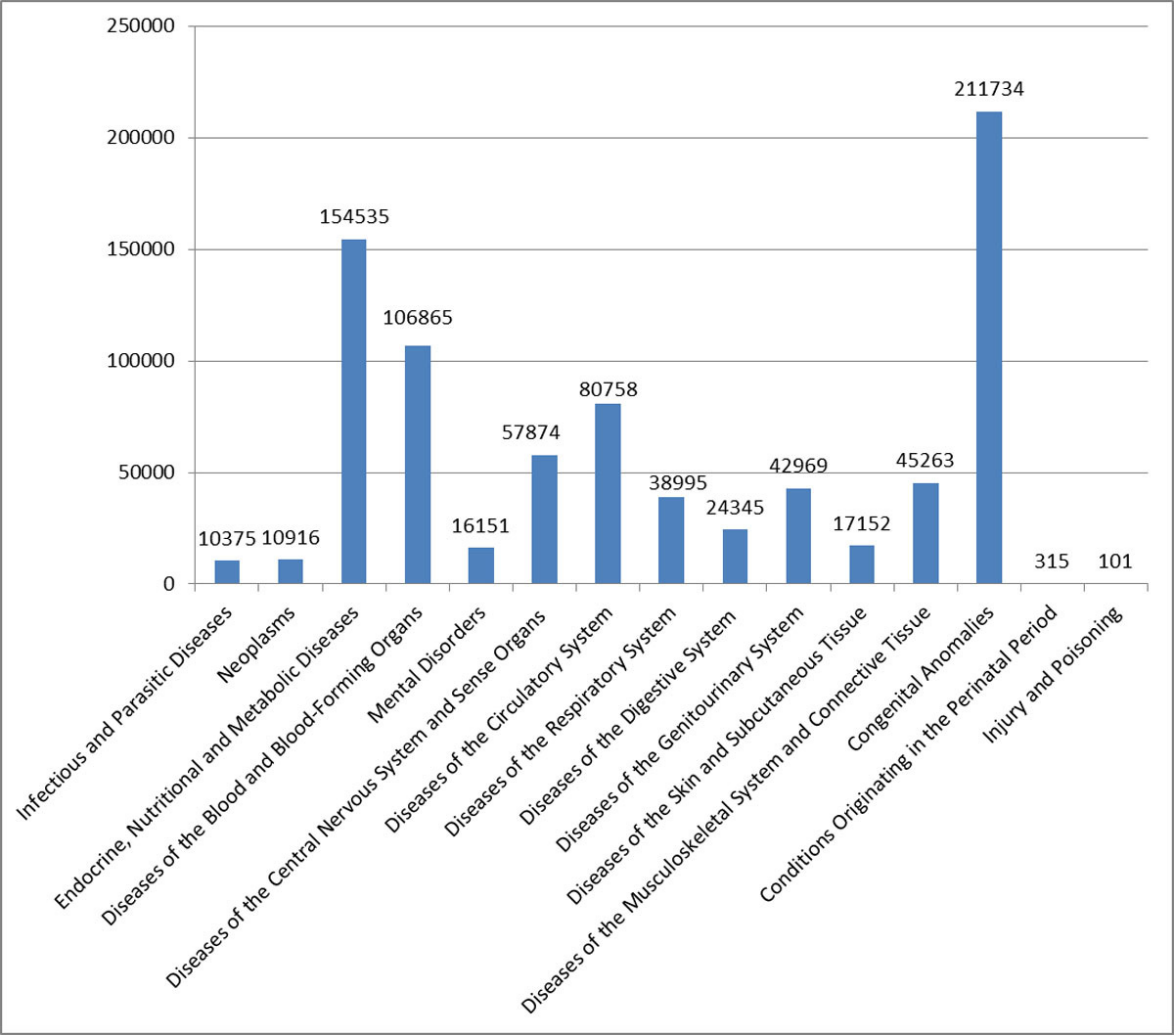
Number of reported RD cases by ICD chapter

Data representing 80.2% of the Spanish population have been initially communicated to the central data repository during the pilot study (Figure 1). A total of 824,399 RD cases have been detected. As an example, RD show 26% congenital anomalies; 19% endocrine, nutritional and metabolic diseases; 13% blood and blood-forming organs and certain disorders involving the immune mechanism; 10% diseases of the circulatory system (Figure 2). Practical problems detected in the pilot study have been discussed and fixed. Final patient recruitment has already started and it will include RD cases detected from 2010 to 2012.

## Conclusion

In summary, National Institute of Rare Diseases Research and Regional Health Departments of Spain are working together towards a harmonized RD patient registration. The Spanish experience could be a model for other countries with complex political and administrative structures which, in order to carry out a national RD registry, will require the standardization of criteria, data harmonization and coordination between regions.
